# "The Hospital" Nursing Mirror

**Published:** 1897-02-20

**Authors:** 


"The Hospital" Nursing Mirror.

Being the Nursing Section of "The Hospital."

[Contributions for this Section of "The Hospital" should be addressed to the Editor, The Hospital, 28 & 29, Southampton Street, Strand, London, W.C., and should have the word "Nursing" plainly written in left-hand top corner of the envelope.]

### News from the Nursing World.

### THE VICTORIAN ERA EXHIBITION.

The Victorian Era Exhibition, which will open at Earl's Court in May, bids fair to be worthy of its name. The Duchess of Devonshire presided over a meeting of the Women's Work Committee at Devonshire House the other day, at which the formal arrangement of various sub-committees was settled. Lady George Hamilton has undertaken to look after the nursing and philanthropic section. An exhibit from the Queen's Jubilee Institute for Nurses will have a prominent place at the exhibition.

### MRS. GOSLETT ON TRAINED NURSES.

Mrs. Clare Goslett, a lecturer on sanitation and hygiene, undertook an exhaustive criticism of nurses and nursing recently at Torquay, when she addressed her audience upon "Nurses à la Mode." The utterances of irresponsible people on these matters need little comment; but there are two points upon which a word might be said. Firstly, "Twenty years ago," said Mrs. Goslett, "there was not a nurse institution in the country," a statement inaccurate on the face of it. Mrs. Fry's Bishopsgate Institute for Nurses was established in 1840; in 1848 a home for training nurses was started by the then Bishop of London, Dr. Todd, of King's College Hospital, and Mrs. William Morrice known then and still as St. John's House. In the sixties many nursing institutions sprang into being, notably perhaps the East London Nursing Society, the Manchester and Salford Sick Poor Nursing Institution, while the Metropolitan and National Nursing Association has existed since 1875. Mrs. Goslett should have got up her facts with more care. The second point concerns a remarkable invitation to "heads of households" to obtain sufficient "training or reading to be able to supervise nurses and to keep them in their proper position"! Will not the medical man have a word to say on this matter? Perhaps he is to be supervised and kept in his position, too!

### SOCIETY OF TRAINED MASSEUSES.

The Society of Trained Masseuses held one of their usual pleasant social gatherings last week. After the presentation of certificates to the successful candidates at the last examination, an address was given by Miss Wilson, hon. secretary to the Workhouse Infirmary Nursing Association; and Miss Gethen told some of her popular "Hospital Stories." Mrs. Burnett Smith (Annie Swan) was amongst the guests.

### A NURSING DEPARTMENT FOR THE L.G.B.

Rumours are in the air of contemplated changes in the Local Government Board, and the possible establishment of a new and separate children's department. There is no matter which needs more immediate attention than the present condition of nursing under the Poor Law, and no step more urgently needed than the establishment of a nursing department, or sub-committee, composed of men and women qualified to judge of the needs and necessities of the sick and of the nurses who tend them. No rearrangement of the Local Government Board will be satisfactory which does not include this long-deferred and patiently waited for reform. The nursing question in the smaller workhouses is daily becoming more and more fraught with difficulties and impossibilities. The position of the trained nurse under the untrained matron grows more intolerable week by week, and something must be done, and done soon, to remedy a state of things which is no credit to a civilised country. For the efficient carrying out of the needful reforms a proper department must be organised. Now is the opportunity for facing this very serious question, and settling it once and for all.

### THE PRIVATE NURSE'S GRIEVANCES.

A study of the letters from private nurses which have been elicited by Lady Priestley, some of which we have published in another column during the last few weeks, provides much food for thought, and throws a great deal of light on the whole question. One thing appears to us to be very evident—those women whose equanimity can be disturbed because they sometimes find themselves in the houses of people who do not accord to them "the courtesy and deference" due to ladies "accustomed to the refinements of a well-appointed home"—as one of our correspondents put it —should give up nursing altogether. Every word uttered by these nurses, who are so anxious to label themselves as "gentlewomen" shows that they have no worthy conception of the dignity and honour of the noble profession to which they belong, nor of the true and only real meaning of the words "gentlewoman," and "lady." For this, let every woman read Mr. Ruskin's grand words in the latter part of "Sesame and Lilies," and try, if she may, to make them her own. Truly do nurses on this point seem to be abdicating their "majesty" of helpful womanhood to "play at precedence with their next-door neighbour"! We are no defending the vulgarity with which private nurses have to come into contact—doubtless unpleasant enough—but the vulgar need nursing, as well as the refined, and require educating more. By this attitude of mind, this looking out for, and resenting of, slights, what opportunities are not private nurses throwing away!

### SECTARIANISM AGAIN.

Some of the supporters of the Royal Victoria Hospital at Bournemouth have been lately much disturbed by statements which have been made to the effect that only "ladies by birth and members of the Church of England" are eligible as probationers in that institution. Such distinctions of creed are always to be regretted, and are indeed indefensible in institutions which are supported by all classes of the community irrespective of religious differences. Now that the attention of the committee has been drawn to the matter it may confidently be expected that this undesirable restriction will be promptly put an end to.

### THE QUEEN'S REIGN.

FROM all over the country we receive news of projects many and various for the commemoration of the Queen's long reign, and it is certain that Her Majesty herself will be well pleased that the form chosen is almost universally one for the benefit of the sick. Amongst the latest schemes proposed may be mentioned the erection of a convalescent home for women by the County of Surrey, of which the Duchess of York has consented to become president; the addition of a wing to the Peterborough Infirmary; and the establishment of a cottage hospital at Gainsborough.—Salford has decided to celebrate the Queen's reign by the establishment of a central nurses' home in connection with the Manchester and Salford Sick Poor and Private Nursing Institute, affiliated with the Q.V.J.I.

### NURSING AT THE BOW INFIRMARY.

THE committee of the Bow Infirmary have now filled up the vacancies on the nursing staff by engaging private nurses from Guy's Hospital and the Nurses' Co-operation, and generally making an addition of twelve staff and assistant nurses.—At a recent meeting of the Board of Guardians, when the question of appointing an assistant medical officer to the infirmary came up for discussion, a proposal that women candidates should be eligible for the post was unanimously adopted.

### MR. BANCROFT AT CHELSEA.

MR. BANCROFT gave his last reading of Dickens' "Christmas Carol" yesterday afternoon on behalf of the Chelsea Hospital for Women at Stafford House, kindly lent for the occasion by the Duke and Duchess of Sutherland. His Highness Prince Edward of Saxe-Weimar presided. The hall of the grand staircase was well filled, and the reading was received with great applause. At the conclusion, His Highness, thanking Mr. Bancroft on behalf of the hospital, stated that it would benefit to the extent of over £300; adding that the total amount thus handed to charitable institutions by Mr. Bancroft during the past season had reached a sum of £3,000.

### NIGHT NURSING (?) IN WORKHOUSES.

SOME facts elicited at a recent inquest at Newport should be printed, framed, and preserved in the British Museum in lasting memorial of the boasted civilisation of England towards the close of the "glorious Victorian era." A seven-days' old infant was overlaid by its mother in the maternity ward of the Newport Workhouse, and found dead by the day nurse when she went on duty. In reply to the coroner's questions it appeared that there were 133 sick people in the infirmary wards; that they were looked after during the night by "a Mrs. Mountain," with the help of a male pauper in the mens' ward, "but none in the women's ward"; that the wards were ten or eleven in number; that the "nurse" was "a woman about sixty years of age, neither skilled in nursing nor certificated," but with "a good deal of experience in watching the sick"! The medical officer at the workhouse, replying to the coroner's question, "Supposing she were the most certificated and most competent woman in the world, would one woman, even with the assistance of a man, be able to look after 133 patients?" said, "It is an impossible task; it is under discussion at the present time; it is a chronic subject." The jury returned a verdict of "accidental death" (which seems hardly to describe the case), and added a rider that "three or four more night nurses were necessary to look after the patients." How many more of these revelations will be needed before such horrible neglect is made impossible once and for ever?

### FIR VALE INFIRMARY, SHEFFIELD.

THE nursing staff of Fir Vale Infirmary, Sheffield, gave their usual annual entertainment on February 8th to the convalescent patients and inmates, in the dining hall of the workhouse. The nurses' efforts were well rewarded by the appreciative enjoyment of their large audience. The patients in the sick wards, too, have been cheered by a series of concerts given by the Senior Resident Medical Officer (Dr. Sorley) during the Christmas and New Year's season.

### NURSING IN CANADA.

THE annual report of the Montreal General Hospital says: "The training school for nurses continues to keep up a high standard of excellence under the direction of Miss Livingston. The period of training has been extended to three years, an arrangement which is of decided advantage to the hospital in giving it the services of nurses for a third year. There have been thirteen graduates during the year.

### SHORT ITEMS.

THE Queen has been graciously pleased to confer the decoration of the Royal Red Cross upon Sister Mary Aloysius, in recognition of her services in tending the sick and wounded. — The Grand Duchess Serge of Russia, who, like her mother, the late Princess Alice of Hesse, has always taken much interest in nursing matters, has consented to become President of the Red Cross Sisters at Moscow. —The new nurses' home at Wandsworth Infirmary is to contain eighty bedrooms. — Lady Londonderry presided over the annual meeting of the Newtownards District Nursing Society, at which a most satisfactory report was adopted. Cordial mention was made of Nurse Love's excellent work.—Two nurses have been dismissed from the Bow Infirmary for "misconduct," brought to the notice of the committee by the matron, and admitted by the nurses.—A performance of Handel's "Samson" will be given in the Shoreditch Town Hall, on February 25th, by the North-East London Choral Society, in aid of the funds of the Haggerston and Hoxton District Nursing Association.—Miss de Pledge having retired from the editorship of the *Nurses' Journal*, the conduct of the journal now rests in the hands of the Editorial Committee.—Ada Florence, eldest daughter of the late Rev. H. H. Monro, M.A., Rector of Sherwood, Naini Tal, N.W.P. India, a nurse at the City Hospital, Coventry, was married on the 10th inst. to Charles E. C. Grant, youngest son of the late George Grant, of Lanark House, Upper Norwood. The wedding took place from the hospital.— We announced last week that Miss Hope had been appointed Matron at the Cornwall County Asylum, Bodmin. It should have read "matron of the Private Patient Block." Miss Vicary has been matron of this asylum for many years.—It has been decided that the new club for nurses and associated workers in "The Hospital" Building, 28 and 29, Southampton Street, Strand, W.C., shall be called the "Victoria Commemoration Club," to connect it with the sixtieth year of the Queen's reign.

### Lectures to Surgical Nurses.

By H. A. Latimer, M.D. (Dunelm), M.R.C.S. of Eng., L.S.A. of London, Consulting Surgeon, Swansea Hospital; President of the Swansea Medical Society; Lecturer and Examiner of the St. John Ambulance Association, &c.

### I.—THE ART OF NURSING—NURSES OF THE PAST AND PRESENT CONTRASTED—ATTRIBUTES OF A GOOD NURSE—THE USES OF THESE LECTURES—HOW MEDICAL MEN INVESTIGATE DISEASE.

I think it will be well in the few introductory remarks which I shall be making to this systematic course of lectures on "Surgical Nursing" if I dwell on the fact that nursing has become one of the arts. A good definition of an art is that one which says "it is the employment of means to the accomplishment of some end, directed by knowledge." Time was when a nurse, as you are all aware, did not depend upon knowledge directed by previous training, but upon a haphazard method of her own, acquired during the course of her daily work. Things have altered so much now, and the value to surgeons and physicians of the trained nurse has become so widely known that the vocation of a nurse has become more and more frequently adopted as its usefulness has become recognised by the community. In the old days, before nursing was systematised, here and there one met with a woman who might have been described as a born nurse; but, for the most part, nurses were so indifferently equipped for their work that they often did more harm than good in their attendance upon the sick. Dickens, by his portrayal of these nurses under the name of "Sarah Gamp," did more, perhaps, to banish this class from the scenes than can well be imagined. She was oftentimes good-natured it is true, but this good nature of hers was mixed with an amount of prejudice and of self-reliance which prejudiced and unskilful persons possess to a remarkable degree. She was self-reliant without knowledge, and, not understanding the reasons which the medical man had for giving certain orders, was very prone to neglect those orders to the detriment of the patient in her charge, and to substitute a treatment of her own when the doctor's back was turned. Let us contrast this style of nurse with one who has received a systematic training. If the latter has chosen her vocation aright she will be gentle and sympathetic towards her patient, she will have acquired a skilfulness of method in handling him, she will be thorough because she performs her duties in obedience to directions which she is able to understand, and, for the same reason, she will not be fussy, because she will have a reliance upon herself which always goes hand in hand with knowledge. She will not be a gossip, blabbing of the cases which she has attended, with a view of magnifying her office; she will be considerate in small matters, and unostentatious in her work in the sick room. On this matter of not gossiping about her cases, I think it will be right if I dwell for a few moments. You cannot be too careful at all times to avoid giving details to the sick of your attendance upon other sick persons, for, although at the time that you are talking to them they may appear interested in what you are saying, you must remember that a sick person is a nervous person, and that during the long hours of want of rest and of disturbance of the mind and body which accompany sickness, he is apt to dwell upon your recitals of other illnesses which you have seen, and to imagine himself to be suffering from ailments of a like nature.

You will see as you go through your work how many diseases have symptoms in common; how it is the special symptom here and there which marks the individuality of a special complaint, and as the symptoms are oftentimes so common in different diseases, the sufferer is very apt, on hearing some of them described, to think that the description fits his own case. Whilst on this point of gossiping, I think I shall do rightly in warning you against talking of the management of other houses and households in which you have been placed. The nurse should be a person who can be thoroughly trusted to safeguard the secrets not only of her patients, but of the households in which she has done her work. There is another side of her character to be regarded, and that is her conduct towards the medical man who is in attendance. She should be at all times loyal, never doing aught to detract from the esteem in which he is held in the house where she is employed: and she should remember that she stands there to do her best for the patient, and she will be doing this best if she upholds at all times the authority of the surgeon who is visiting there. I have known, in not a few cases, nurses who have had a predilection for one man over another, and, showing their predilection, they have undone much of the good they were placed there to do by disturbing the patient's mind and displacing the confidence he has had in the man who is doing his best to cure him of disease. She must be cleanly in all her habits, and in the care of all instruments or appliances in her charge; also, as she is in attendance for long periods, she should become a skilled observer of symptoms, of their continuity, their variation in type, and of the advent of new ones. She should be ever ready to note changes of position, appetite, pain, colour, and such like things; she should be punctual in carrying out the orders entrusted to her, in administering medicines, and giving foods; no detail should be too trivial for her observation, and in this regard I would mention that she will acquire the art of observation to its fullest extent by cultivating a habit of making notes in a book she should carry with her. This record, when given to the surgeon, may be of the greatest importance to the welfare of the patient: suppose, for instance, the patient is suffering from some surgical disease, and at one time he has a shivering; this will note a change in his condition, possibly the formation of matter, possibly the appearance of an attack of inflammation of the lungs, and when reported to the surgeon it at once places him on the look-out for some new complication in the illness, and gives him a clue to a further examination to detect the same.

The use of these lectures will be to explain why and wherefore the surgeon issues his orders to you. If you make full and intelligent use of them, and if you practise to its fullest extent the opportunities of practical work which are afforded you in the wards of a hospital, under the direction of the sisters who are placed in charge of those wards, and of you, you cannot fail, having an aptitude for it, to become useful and skilful nurses. They will render you what all medical men desire to see you, intelligent co-operators with them in the great work of relieving pain and distress, and curing the sick. You will find that if you adopt a system in your work this in time will make the same easy to you. Medical men are taught to systematise their work in learning their profession. There is no heaven-born method by which a man is able to see that another is suffering from a definite disease. It is true that after long years of experience the medical man is often enabled by a kind of intuition to diagnose the malady of the patient who presents himself to him, but the man who conducts his work on these lines is very prone to make mistakes, and to overlook things of great importance. To guard against this I would have you observe symptoms in the same way that a doctor is taught to investigate them, taking parts of the body one by one, after knowing the functions of those parts, and then applying the knowledge you have of those functions to the perturbed workings of the same.

### Post-Graduate Clinics for Nurses.

By a Trained Nurse.

### III.—THE EVOLUTION OF THE PRIVATE NURSE.

In the reformed curriculum of our English Training Schools—and reform is coming in due process of time, being hindered rather than helped by violent agitation—some provision must needs be made for her systematic training in the duties pertaining to private work before the nurse can be regarded as thoroughly efficient. The first step towards this has been taken in the establishment in a favoured few of our hospitals of courses on invalid cookery, a branch of nursing which should be recognised as being quite as essential to a thorough training as is a capacity for taking temperatures and doing an artistic "figure of eight." Here again the American hospitals are in the van. I remember a visit to the charming diet kitchen of the Charity Hospital on Blackwell's Island, New York, this being a hospital occupying much the same position as one of our English workhouse infirmaries. The culinary arrangements were charming, and one could not help appreciating the wisdom which made the care and preparation of all the "extra diet" for the sick a part of the ordinary routine of training. The delicious beef-tea, the tempting custards, and meat essences prepared by the nursing staff were striking contrasts to the inferior and badly-cooked food which in many of our poor-law infirmaries is thought good enough for "pauper inmates." The patients of the Charity Hospital include the worthless of all nations, but, when sick, they are splendidly cared for, and their cooking done by intelligent women with trained minds and hands, who thoroughly recognise that the diet kitchen is of equal—or perhaps in some cases of more—importance than the dispensary.

In Chicago, too, service in the diet kitchen of the Training School is considered essential before a nurse is certificated, and there, in many Institutions, such a refinement as cooking by electricity has been introduced, so that the nurses are able to go through their cook-training in very dainty fashion, without the dustiness and over-heating which is the natural accompaniment of stoves and coal-fires.

Instruction in massage is another important branch of nurse-training, and this is included in the curriculum of an American trained nurse, and is, of course, of the utmost value. For while it is not necessary for every trained nurse to be at the same time a masseuse, and, for the matter of that, general massage is too fatiguing to combine with nursing, occasion frequently arises in the course of a case when, for instance, abdominal massage may be necessary for habitual constipation, or the rubbing of a limb may be ordered after rheumatism or minor surgical accidents. And, of course, it is a great advantage to doctor and patient when the nurse knows how to perform these duties in a competent manner.

The application of electricity is another refinement in the art of nursing which is taught as a matter of course in the Training Schools of the United States, where electrical treatment is very common not only in the cases of neurasthenia, which the climate and high-pressure of American life produce in such miraculous numbers, but also in surgery.

It is true that the battery and electrical treatment do not form a factor of much importance in English therapeutics, and that, when used, many practitioners prefer to apply it themselves. But, unquestionably, the use of electricity in treatment appears to be necessary, and the management and application of a battery being so simple and uncomplicated a matter, there appears to be no reason why the average English nurse should not learn its use, as does her American sister. It is evident, too, that nurse "treatment," in every sense of the word, occupies now, and will eventually occupy to a still greater degree, an ever-increasing importance in therapeutics. And with the development of scientific requirements, the nurse, too, must develop.

In discussing the evolution of the private nurse it seems hardly necessary to bring further proof that the ordinary curriculum of an English hospital, while it unquestionably affords an admirable training in the theory and practice of nursing, gives no special teaching for the benefit of those of its nurses who elect to take up "private work." The officialism and the etiquette of a hospital, its rules and regulations, form as it were a kind of supporting splint for the staff. If any doubt or difficulty arise there is always someone in authority to whom appeal is possible. Both Sister and Matron are ever ready to give advice to inquiring pros. and puzzled "staffs." But there is never an advantage without its corresponding drawback, and this necessity for appeal to other judgments, and the finality of a decision once pronounced by a superior official tend in two directions. The nurse withdrawn from such supporting authority may display the fault of too little self-reliance, or—and this, so far as private nursing is concerned, is much the worse—she may develop an atmosphere of officialism and red-tapeism which private patients will almost invariably resent.

It is quite clear from the manner in which patients and their friends discuss and criticise the hospital nurse in private practice that there is much which is faulty in the system of training those who intend to work under conditions so different as are the conditions of a hospital ward and a private sick-room.

"During the whole of my five weeks' illness nurse never once let me have a quiet half-hour's chat with my husband. She hadn't the tact to leave us together, and we thought she would be offended and think I wished to complain of her if we asked her to leave the room. Of course, there were many things we wished to talk over without the presence of a third person, but she was so kind and pleasant we did not like to hurt her feelings. So we just bore her constant attendance, but we *were* so glad when she left the house."

This nurse, fresh from hospital, did not realise that the surveillance of ward visitors and the danger of contraband articles being smuggled in had no relation to private work. Hence, although an excellent nurse and a very pleasant woman, she created a feeling of opposition, almost amounting to dislike, on the part of her patient, entirely arising from a too conscientious conception of her duty. Again, during a serious illness in the familyof one of my friends—illness extending over some fifteen months, and necessitating the attention of two nurses, one for day and another for night duty—I gained an insight into some of the pitfalls a want of knowledge of the world and its ways may open up for a nurse.

One of the nurses complained seriously to the head of the household, whose wife was the patient, because she and her sister nurse were not invited to dine with the family. Now, the household is a fashionable one, and part of the time during which the nurses were in attendance was during the London season, when my friends not only gave frequent dinner parties, but had guests constantly coming in to dinner, and the addition of the two nurse-strangers to the table would have been distinctly inconvenient and out of place. So that, as a mere matter of common sense, such an expectation on the part of the nurses was perfectly unreasonable, and one would have thought to dine under such circumstances would have been as uncomfortable to the nurses as it would have been restraining to the family and their guests. But, although every possible consideration was shown them in the matter of meals, the nurses felt aggrieved, and nothing would satisfy them wholly short of "dining with the rest," a point which, however, they did not succeed in carrying, although considerable friction resulted from their agitation.

### Seven Weeks in a London Fever Hospital.

By a late Patient.

### I.—FIRST IMPRESSIONS.

Probably few people (save ex-patients) have any idea of the inner life of these institutions. Yet they play so large and useful a part in our great metropolis that a detailed sketch should prove interesting. Let me try to give a picture of my stay in the North-Eastern Fever Hospital at Tottenham, and afterwards at the Northern Hospital, Winchmore Hill.

I was living in a floor of rooms in the Holborn district when I fell ill, first with a sore throat, afterwards with a rash. I had been in bed several days when the doctor announced that I had scarlet fever.

I had two courses before me—isolation in my rooms or a move to hospital. My slender means would not bear the combined expense of doctor and nurse for myself, and a separate establishment for my wife and child; this, and the evident nervousness of other people in the house, decided for the latter course, and the request for removal was sent to the sanitary inspector. Next morning I heard a vehicle stop in front of the house, and, a minute or two later, the voice of my wife in conversation with a lady whom I rightly surmised was the nurse in charge. In another moment a pleasant-looking woman, wearing a uniform of blue and white linen, with a white apron, entered the room. Drawing forth a sheet of paper, she asked a few questions—my name, my wife's name, the date of symptoms appearing, &c. These duly recorded, she produced a large flannel dressing-gown and several blankets, and in two or three minutes I was divested of my own clothes and attired in the dressing-gown. I was about to rise, thinking myself ready for travelling, but was gently restrained by the nurse, who proceeded to swathe me in the blankets after the fashion of an Egyptian mummy, till I could move neither hand nor foot. Then, telling me she would send someone to fetch me, she glided from the room.

In a few seconds a stalwart man, in appearance something like a railway guard, appeared, and, grasping me round the waist, carried me downstairs (leaving but just time to shout a good-bye to my wife) and deposited me in the waiting ambulance, a small closed vehicle something between a private omnibus and a prison van, and painted partly dark green and partly yellow. The inside was half filled with a kind of sliding couch or bed, fitted with an air pillow. The other side was clear with the exception of the nurse's seat by the head of the couch. The doors were closed, and we rolled gently away, followed by the astonished glances of a juvenile crowd which had quickly collected. At first I occupy myself with trying to make out our route, but the windows are of blue glass, and I can see but little. After passing St. Pancras Station I lose my reckoning, and lie back on the couch reading an evening paper which the nurse has kindly bought for me at my request.

The journey takes about an hour. Occasionally I try to talk to the nurse, but she is not able to tell me our whereabouts, and the movement of the ambulance does not favour conversation. Suddenly the nurse says, "We are there now."

I have a swift view of spacious grounds intersected by a number of long, low, wooden buildings with corrugated iron roofs. The attendants lift me out of my chariot, and I am carried to one of these buildings. The door opens, and I find myself in the receiving room. It is a small, square, light room, with a couch in the middle and a washstand on one side. The only occupant of the room is a fair young lady in a similar uniform to that of the nurse of the ambulance. She comes forward, and, helped by my nurse, they undo my "mummy" wrappings and dressing-gown, and cover me with a large red blanket. After a few minutes' waiting the doctor arrives—looks at my chest and throat, receives the paper with my name on from the nurse, and writes on it his diagnosis of the complaint. I am lifted on to a stretcher upon the ground, and while waiting for a few moments, the door opens and the next "case" arrives. It is an infant which is now brought in and placed—a small, motionless bundle—on the couch I have just quitted. The nurse in charge of the room cries, "Oh! look at this tiny baby," and bends over it with a look of quite tender solicitude—if it had been her own child she could not have shown a more affectionate interest. Truly my first impressions of the North-Eastern Hospital are favourable ones! But now the men lift my stretcher, carry me out of the room, and into a strip of planked flooring about four feet wide and ten yards long, with a roof which is supported on upright poles. These wall-less corridors form a covered approach to the different wards, the long wooden huts I have seen. That into which I am taken is a one-storey building about 150 feet long by 20 feet wide. The roof is of bare planks, the floor ditto, relieved only by the windows which run all down both sides, and the few pictures (mostly of the illustrated paper supplement order) which, framed and unframed, hang over some of the beds. Everything is simplicity and plainness itself, but light, airy, and cheerful. The beds are in rows on each side of the ward, the heads of each against the wall, between the windows. The middle of the long room is partly filled by four plain wooden tables bearing a few plants and the medical appliances. A few wooden chairs, one or two caned-backed easy chairs, a stand beside each bed, and a few pink chintz screens make up the furniture of the ward.

The white-quilted beds are occupied mostly by boys, some lying down quietly enough, others sitting up in bed in red flannelette jackets. The nurses seem to be three in number—a head or "charge" nurse and two assistants. They all wear the uniform I have already noticed, the "charge" nurse being distinguished by a flowing white streamer while the others have caps.

Most of this I noticed in a few rapid glances while a hasty colloquy ensues between my bearers and the nurses. As a result I am marched nearly to the top of the ward and placed on a chair, where I sit wrapped up till they have put clean sheets on a bed, in which I am then placed. After lying still for a couple of hours, I grow hungry. Visions of dinner float before my eyes! I ask the charge nurse what I can have to eat. She tells me I can only have bread and butter now (I am too late for dinner) and milk puddings for dinner. This is alarming—uncomfortable. I ask what shall I have to drink. She answers, "Either milk or lime-juice." Great Guinness! I am seriously frightened now. She sends for some milk, which I drink, and lie back with a heavy heart. "Why you poor man," says the head nurse, smiling amiably, "don't you know you have to be starved at first? You are on low diet." Then I find out there are three classes of diet—"low," "middle," and "full." I am on the first. I pass in view all my past sins, and try to think for which I am visited with this affliction. At four o'clock the nurses come round with tea and bread and butter. I eat a little, and then have another look round the ward.

There are four or five young men besides myself—the rest boys. None of them seem seriously ill. Presently a nurse kindly brings me the literary stock of the ward—a bundle of *Tit Bits*, *Pearson's Weekly*, *Graphic*, *Illustrated News*, *Strand Magazine*, &c. These I read by gas-light till nine o'clock, when, by inexorable rule, the gas is turned down low, only one bracket being left on—that of the night nurses, who have now relieved the ordinary staff. They come on duty at eight o'clock, and have to maintain a sleepless vigil till morning. With difficulty I drop off to sleep at this early hour, and so ends my first day in hospital.

I am roused next day by considerable bustle. The light streams brilliantly through the long row of windows. I find it is six o'clock and bed-making is going on. The nurses come to me, and I wrap myself in a blanket and sit in a chair while they make the bed, and then get back again. They are most particular about the neatness of the beds, these nurses, and declare I am very untidy. Breakfast comes, and such of the patients as are well enough to get up help the nurses in waiting. Tea, and bread and butter. After breakfast the ward is carefully swept up, basins of water are brought to us to wash in. At half-past ten or eleven o'clock the doctor arrives. He looks at the diagnosis of my complaint (now hung above my bed), also at my tongue and chest.

Emboldened by his opinion that I have a slight attack only, I ask for a change of diet. "Can I have a few tomatoes?" "Well, yes, I think so." He takes down the paper, writes on it, and passes on. I eagerly turn to it and find he has written "middle diet," and made a note "tomatoes." For this relief much thanks. I ask a nurse what middle diet is. "Oh! fish for dinner, one egg with breakfast, and salad." And now the morning paper ordered by my wife arrives, and I read that and the few magazines till dinner. This meal is at twelve o'clock. I anxiously wait the approach of "middle diet." Presently a young nurse comes along and gives me a plate of fish, with potatoes, cabbage, and bread. I receive it with dog-like gratitude, and my heart warms towards this young lady who has given me my first "square meal."

### Prince Alfred Hospital, Sydney.

(FROM A CORRESPONDENT.)

ENGLISH readers may perhaps like to hear something of the way the Christmas just passed was celebrated at this colonial hospital, which has obtained quite a reputation for the successful efforts of the staff at that season to make high festival as far as possible for patients and nurses. The wards were prettily decorated, the patients greatly appreciating the nurses' endeavours to give them a happy time, and all who were well enough thoroughly enjoying the treats provided for their benefit. The committee supplied jellies and cakes, and presents of fruit were received in sufficient quantities to give a share to all who were able to take it; while grateful ex-patients sent many good things to the wards where they had themselves been nursed. The plum pudding, which in faithful adherence to English custom is always part of the *menu* at Prince Alfred's Hospital, was done full justice to.

The children had a charming tree presented by a number of ladies, who came to the hospital on Christmas Eve and distributed their gifts. On Christmas morning every child found two presents under his or her pillow, sent by Miss H. James and Mrs. MacCormick. Miss James has for many years collected toys and other gifts from her friends for the patients at Christmas time, no one but herself, perhaps, knowing how much trouble and time has been thus expended, but she takes real pleasure in giving pleasure to others, and her pretty gifts are more than welcome. Two old patients sent quantities of lovely flowers.

On Christmas Eve, about a quarter to eleven, the nurses and sisters, twenty-eight in number, accompanied by Miss McGahey and some of the medical staff, started on a carol-singing round, the honorary organist to the hospital, Mr. Howe, accompanying the singers on an American organ. It was a striking scene; the hospital and grounds looking beautiful under the light of a glorious moon, the breeze carrying the sound of the familiar hymns and carols quite a long distance. The singers took up their positions on the balconies of the various pavilions, and the patients listening to them thought it was "like heaven." A special service was held in the chapel on Christmas Day. All the festivities went off with great success, and were thoroughly enjoyed by everyone in the hospital.

### The Midwives' Registration Bill, 1897.

THE Midwives' Registration Bill which was introduced into the House of Commons by Mr. Tatton Egerton on February 9th differs in some points from those which have preceded it. In it the term midwifery nurse so much insisted on by some is entirely dropped. It is a Bill for the registration of midwives. In the definition of a midwife all reference to attendance on "natural labour" is also omitted, and if the framers of the Bill had been content to define a midwife as a woman who undertakes to attend cases of labour, their definition would at least have accorded with facts. Unfortunately they have added the words "in accordance with the regulations to be laid down under this Act," and so raised all sorts of questions as to the interpretation of section 3 (1). Lord Balfour's Bill as amended in the House of Lords left the English language to take care of itself. There is no necessity to define a midwife any more than in a muzzling Act there is any necessity to define a dog.

As in Lord Balfour's Bill, the whole purport of the Bill is to prevent women using the title midwife, or any title implying that they are registered under the Act. There is no attempt made to restrict the practice of midwifery by unqualified persons. After a certain date no woman is to take or use the title of midwife, &c., unless registered in accordance with the provisions of the Act, but any woman in *bonâ fide* practice as a midwife for two years before the passing of the Act, or who has held a certificate in midwifery from the the Royal College of Physicians of Ireland, or from the Obstetrical Society of London, or such other certificate as may be approved by the Midwives' Board, may be registered if she sends in her claim within two years of the passing of the Act.

The Midwives' Board is to consist of eighteen persons; twelve being registered medical practitioners, appointed by various bodies, including the Midwives' Institute, and six being persons appointed by the Lord President of the Council. This Midwives' Board shall, among its other duties, "frame for approval by the General Medical Council rules for regulating, supervising, and restricting within due limits the practice of midwives." This is the really important clause, and the position of the registered midwife of the future will entirely depend upon the view taken by the Midwives' Board and the Medical Council as to the scope of these rules.

"Every local sanitary authority shall, on the passing of the Act, appoint its medical officer of health, or other registered medical practitioner or practitioners, as the local supervising authority," but the power of this authority does not seem to extend far beyond investigating charges of malpractice, &c., and reporting to the Midwives' Board.

On the whole the Bill seems to have in it certain elements of success—so far as getting through Parliament is concerned. This, however, is attained by putting off the evil day. The rub comes in the framing of the rules under which the midwife is to work.

The real problem, however, the only matter that is of any general interest, or of any real importance to the people, is left untouched by the Bill.

The real excuse for legislation on this subject is that at the present time any woman, however ignorant, drunken, or immoral she may be, may take to midwifery as a means of eking out a living. This is a great scandal, and it is this that is the cause of so much loss of life. And this is untouched by the Bill, in which there is not one word to prevent anyone who chooses practising midwifery as much as she likes or in any manner she likes, on the one condition that she does not take the title midwife.

### Everybody's Opinion.

[Correspondence on all subjects is invited, but we cannot in any way be responsible for the opinions expressed by our correspondents. No communication can be entertained if the name and address of the correspondent is not given, or unless one side of the paper only is written on.]

### LADY PRIESTLEY AND MEDICAL NURSES.

"E. C. D." writes: In all the correspondence in your paper, to which Lady Priestley's article in the *Nineteenth Century* has given rise, nobody has contradicted one inaccurate statement made by her. This may be due to the fact that the statement in question refers to a matter of more interest to the female medical student than the nurse. To quote from the article, "It would be an impossible drop for a woman accustomed to the excitement of hospital life . . . to enter the gates of the female school of medicine, and walk the wards of a hospital managed solely by women; and this she would have to do before she could pass into the world a fully qualified doctor." As a matter of fact, several medical schools in the United Kingdom and the Colonies are open to both men and women; and in towns where, as in London, women have their own medical school, they gain their clinical experience in a general hospital for both sexes, where the physicians and surgeons are all men.

A Trained Nurse writes: If Lady Priestley wishes to see herself conspicuous in print she might have found a more kind and womanly way than her late attack on hospital and private nurses. Surely, as a class of hard-working women, who in private nursing are often on duty seventeen and eighteen hours out of every twenty-four, our fee of two guineas weekly is very hardly earned. I belonged for some years to the second oldest private nursing institution in London, and both in the West-end and large country houses I have had many trials and hardships to put up with. I have returned "from the tension and anxiety of a very serious case, the petty worries of patient's friends," mentally and physically weary and sick, when the relief of being off duty, free to wear private dress, and go to some theatre or concert has been inexpressible. As we have seen from Lady Priestley's late tirades, there are ill-minded women in every station and class. I have also met some grateful and kind people while private nursing, and, like a great number of nurses I know, can count among my most dear and true friends the wives, fiancées, and sisters of young gentlemen I have had for patients. Surely this and many other things show how utterly untrue are many of the statements hurled at us by Lady Priestley; and as a body of hard-working and conscientious women, hospital and private nurses can afford to smile at Hall Caine's utter failure to appear as a modern Dickens—for surely this must have been his aim when he voluntarily took upon himself to expose, as he considered it, a public wrong in his novel.

"Sister L." writes: As a trained nurse of several years' experience at home and abroad, I should like to say a few words with regard to Lady Priestley's attack upon nurses of to-day. In her letter she shows considerable want of knowledge in the detail of the nursing world in general, and from its tone one cannot help feeling that she writes as one having a personal feud to avenge with some of our weaker members. Possibly she has had the misfortune to meet with some of those strange and doubtful characters, dressed up and passing for trained nurses, who are cast-off weeds from our hospital gardens, calling themselves nurses. Alas! there are hundreds of such amongst private nurses. A little learning is a dangerous thing, and these women, who have often only picked up a few months' smattering, will do and dare things in a doctor's absence that no trained nurse would presume to do. They may cavil at a doctor's orders before a patient, but no real nurse will so far forget her own dignity as to lessen the authority of her superior officer, for she knows that often half the cure depends on the faith the patient has in his or her doctor and his treatment. What we learn in the hospitals only shows us how much more there is to learn, and we should not be foolish enough to put ourselves in the false position of thinking we teach better than our teachers. Again, a trained nurse has, if she be a good woman, so much respect for herself and her calling that in all calmer dignity born of modesty, which renders her perfectly fitted, even while young—and it may be beautiful—to nurse the youngest man with all the more gravity of bearing towards him, as she notes the peculiarities of his character, and most nurses know how to stop any undue familiarities or want of respect in the opposite sex towards them. *Honi soit qui mal y pense*, is a motto Lady Priestley might often repeat to herself. The nurse she depicts would be kept in no respectable house for twenty-four hours, and consequently in no institution, and I think if she were asked to mention their names and where they nursed it might be the beginning of a great law affair, in which her ladyship might not come off victorious; there are many ladies who like a nurse not to be a gentlewoman for reasons best known to themselves, many of which I know. No nurse can live as a nurse ought on £1 1s. a-week, and lay up for a rainy day even in the Pension Fund, for what is £52 per annum, even if you take no holiday and work all the year round, when you have to pay rent and buy coal, light, clothes, and food when not employed? If Lady Priestley would just try the experiment for one month she would then speak from experience. Then as to the objection to balls and theatres, and out-door games, such as tennis and golf, for nurses, where is the common sense? What should they do with their holidays? A doctor's is a much more serious calling than a nurse's, yet no one raises an objection to their recreations. Many nurses wear their uniforms rather than squander money or get into debt for a fashionable wardrobe for a month's wear, yet these very women, although nurses, would like to have pretty things, and enjoy wearing them if they had them quite as much as Lady Priestley, and they naturally think that as soldiers and sailors (who often see more dead and dying in an hour than any of us in a year or a lifetime) wear theirs, and are not ashamed of it, neither need the nurses be; not that I advocate for one moment going to balls in an indoor uniform, which few women would care to do. It is a pity that it is a woman who is running down her own sex and a profession where so many are trying to do, and doing, good work in their day, of whom good men still write and think kind and charitable thoughts.

### MR. HALL CAINE AS A CARICATURIST.

London Nurse (now far from "The Madding Crowd") writes: I have always felt that there would be much less crime if criminals were treated with silent contempt, as far as the papers are concerned, and neither their names nor their deeds made public. Notoriety, at any cost, is the aim of many. One would have thought that Mr. H. Caine had made himself prominent enough, without his misdirected attempt at chastisement of a profession he knows nothing about. A doctor once told me he always skipped medical details in novels, as they were so absurdly wide of the mark and showed such hopeless ignorance. Why should not we skip Mr. H. Caine's whipping *en masse?* It is absurdly wide of the mark and shows most hopeless ignorance. Treat his book with silent contempt and its notoriety will die of inanition. I am matron of a hospital in the North of Scotland, and have had much ignorant criticism to put up with; but, knowing that I have the confidence of my doctors and managers, I take no notice.

Mrs. Longueville Bedingfeld writes: Having read the correspondence, &c., *re* Mr. Hall Caine's "Nursing Nightmare," may I refer to another less recent novel, which deals with another class of hospital and nurses, "Esther Waters," by George Moore. It contains what I consider most slanderous libels on Queen Charlotte's Lying-in Hospital, and the behaviour of sisters, students, and pupils in that institution, which is openly mentioned *by name*, and not under an alias. As an old pupil at Queen Charlotte's, my blood boiled when reading the description given of the patients, their sufferings, and the inhuman conduct of those whose duty it was to aid the poor women in their hours of trial. When I trained there (in 1878) the kindest care was always given to all patients whether married or single, and not a word was allowed to be spoken or a question asked that could hurt their feelings. Certainly in those days there were neither "sisters" nor male students there. But I feel quite sure that there cannot have been such a retrograde movement in eighteen years, as to make the conduct described in "Esther Waters" possible. What object can authors have in thus libelling the noble institutions in our metropolis. Were they themselves ill, they would fly to skilled medical men and nurses for aid in their sickness, and from whence could these be obtained were it not for the medical schools and nurse training schools of the hospitals.

### THE VICTORIA (COMMEMORATION) CLUB.

NURSE HIRST writes: In the *Daily News* of Thursday last, the 4th inst., I noticed an article on the new Victoria Club—a club for hospital nurses—and as I, as a nurse, thought it might be of use to me, I have been to see it. I was met by the lady superintendent with great courtesy, and shown all over the premises, and I should like to testify to the complete comfort of the place. I certainly think that such a club will be a great boon to our profession, and meet a want that must be felt by many of us, when we have occasion to come to London, as it will be a central place, where we can meet our friends, of either sex, and where appointments might be made to meet would-be employers. I only hope the club will meet with the success it deserves, and this can easily be done by the co-operation of my sister nurses.

### THE MEDICO-PSYCHOLOGICAL ASSOCIATION AND ITS NURSES.

"AN OLD NURSE" writes: May I ask you to kindly give me space for a few lines in reply to Dr. Outterson Wood's observations upon my previous letter in THE HOSPITAL? While thanking him for his "friendly (?) warning," I wish to say at the same time it is not needed, for I have fallen into no trap laid by anyone, neither am I self-interested or inaccurate in my statements. I believe I may lay claim to have a better knowledge of the asylum nurse and her work than Dr. Outterson Wood or any other past or present asylum medical officers. I want the mental nurses to stir up out of their apathy. Why should they be made a tool to advance the "aims and scope" of the R.B.N.A.? This, according to Dr. O. Wood's own words, is part of the purpose of the scheme of forcing them on its register. I will not trouble you with any further correspondence on this subject, but if Dr. O. Wood would like to compare our interest in, and knowledge of, the asylum nurse and her wishes, you are at liberty (if you think well to do so) to give him my name and address privately, and if he desire it, I am quite willing to argue out the matter with him verbally. Before closing my letter, I wish to say I fully endorse "M.D.'s" letter. He seems to know more about the mental nurse's real wants than any asylum doctor who has written to THE HOSPITAL on the subject.

MISS LILLIE WADDINGTON, matron of Bootle Corporation Hospital, writes: Regarding the challenge in Dr. Outterson Wood's last letter, we cannot but consider it a revelation to hear that the Charter of the Royal British Nurses' Association was intended to admit "special" nurses. The statement involves a serious charge against the administrative ability of that Association, in so far that admission has been refused to "fever" and "obstetric" nurses on the grounds that they have not been fully trained nurses. Evidently "circumstances alter cases." Again, we read "it is not hospital nurses who are resisting the admission of mental attendants on the Royal British Nurses' Association, but the narrow-minded policy of a few who are stirring up hospital nurses in opposition." When the scheme to enrol mental attendants on the Charter of the Royal British Nurses' Association became known, trained nurses immediately resented it with indignation, recognising at once that its accomplishment meant the decided lowering of the status of their Association. Agitators were not necessary, the scheme in question explained itself. It is not merely "a clique" who are opposing it, but hundreds of trained nurses, situated all over the kingdom. They resent and object to this intended encroachment upon their rights earned by labour, in like manner as medical men did when dentists demanded registration on the British Medical Association. Dr. Outterson Wood further wishes trained nurses "to join hands in one common sisterhood with mental attendants and welcome them into their ranks, as they too aid greatly in alleviating human suffering." No doubt they do, and so do dentists. It is argued that mental attendants receive lectures and are trained, thus implying that theory and practice are one and the same thing. Experience teaches us, however, that theory is one thing and practice quite another. It is the combination of these two which makes efficiency. Therefore, to be just, let mental attendants undergo training in a general hospital before seeking registration upon an association meant only for trained nurses and not for "specials." Or, if that entails too much hardship, let them follow the example of dentists and register under an association of their own.

### Appointments.

### MATRONS.

HOME FOR INCURABLES, LIVERPOOL.—Miss Soden has been appointed Lady Superintendent to the Home for Incurables. She was trained at the Northern Hospital, Liverpool.

KIMBERLEY HOSPITAL, SOUTH AFRICA.—Miss Agnes Cowan has succeeded Miss Vacher in the post of Matron at this hospital. Miss Cowan formerly held the appointment of matron at the Government Hospital, Vryburg, Bechuanaland.

LANSDOWN HOSPITAL AND NURSING HOME, BATH.—Miss Beatrice Coombes has been appointed Matron of this institution. Miss Coombes received her training at St. George's Hospital, London, being afterwards successively promoted to the posts of ward sister and assistant matron at that hospital. We congratulate Miss Coombes on her appointment.

### Minor Appointments.

BUCKS GENERAL INFIRMARY, AYLESBURY.—Miss Satchwell has been appointed Charge Nurse at this institution. She was trained at Wolverhampton General Hospital.

MITCHAM SCHOOLS INFIRMARY.—Miss Elizabeth Hutchinson has been appointed Superintendent Nurse at the Mitcham Schools Infirmary. She was trained at Salford Union Infirmary.

HULL SANATORIUM.—A lapse in punctuation led to a slight error in the recent notice of Miss Margaret Gray's appointment as Night Sister at this institution. Miss Gray trained at King's Cross Hospital, Dundee, and at Perth Royal Infirmary.

CARDIFF UNION INFIRMARY.—Miss Annie E. Parsons has been appointed Charge Nurse at Cardiff Union Infirmary. She received her training at St. George's Infirmary, Fulham Road, where she was afterwards promoted to the position of staff nurse.

ST. MARK'S HOSPITAL FOR FISTULA, CITY ROAD, N.—Miss Florence Howard Locke has been appointed Charge Nurse at this hospital. Miss Locke was trained at Bolton Infirmary, where she gained a three years' certificate, subsequently being promoted to the posts of staff nurse and sister.

BOLTON BOROUGH FEVER HOSPITAL.—Miss Annie M. Kirsopp has been appointed to the post of Charge Nurse at this hospital. She received her training at Monsall Fever Hospital, afterwards holding successively appointments as charge and staff nurse at Newcastle-on-Tyne and Birkenhead Fever Hospitals.

COLONIAL HOSPITAL, ACCRA, WEST AFRICA.—Miss A. M. Deeks has been appointed by Government to the post of Sister at the Colonial Hospital, Accra. Miss Deeks is a Nightingale nurse, and was trained at St. Thomas's Hospital, where she has since remained as staff and charge nurse for over four years, only leaving to take up her present appointment. We wish her all success.

### The Book World for Women and Nurses.

[We invite Correspondence, Criticism, Enquiries, and Notes on Books likely to interest Women and Nurses. Address, Editor, THE HOSPITAL (Nurses' Book World), 28 & 29, Southampton Street, Strand, London, W.C.]

HINTS ON ELEMENTARY PHYSIOLOGY. By FLORENCE HAIG-BROWN. (J. and A. Churchill. 1896. 121 pp. Price, 1s. 6d.)

In the preface to this little work we are told that it is founded upon notes taken by the two sisters—Miss Helen and Miss Florence Haig-Brown—of the lectures and demonstrations given by the medical officers of St. Thomas's Hospital to the probationers. It is intended rather as a stimulus to wider reading than a manual complete in itself. Much useful information, however, can be gathered from a careful perusal of its pages. Instruction is brightly and lucidly given, and many of the difficulties which beset young beginners are explained and minimised in a manner which is not possible in larger text-books. The chapters dealing with the bones of the skeleton and the circulatory organs are simply and forcibly written with little interesting explanations that will be at once a recommendation to the youthful and at times weary student, whose aim it is to acquire knowledge of these subjects in an easy, straightforward way. The description of the muscular and nervous systems call for special notice on this score, and the chapter on "Inflammation" is one that will be read with the greatest possible advantage by every nurse. The visible symptoms are first described, and then the constitutional symptoms, with the reasons for the same. "Food and its Uses" is another important subject worthy of study. The concluding chapter on "Ventilation" contains many valuable hints on a too often neglected point. The definition that "proper ventilation consists in clean air displacing foul air, constantly and steadily, without chilling the patient," sums up the principles of the matter in a nutshell. We must take exception, however, to so low a temperature as 57° being sufficient for an ordinary dwelling-room, though it is a fault, perhaps, on the right side in these days, when the tendency of the majority is to live in an over-heated atmosphere.

A MANUAL FOR HOSPITAL NURSES. By EDWARD J. DUNVILLE, L.R.C.P., M.R.C.S., Surgeon to the Devon and Exeter Hospital. (J. and A. Churchill, 7, Great Marlborough Street. 119 pp. Price 2s. 6d.)

We heartily welcome the eighth edition of this handy little volume, which is a proof, if one were needed, of its continued popularity with nurses and probationers. So much useful information in a small space we have not often seen, and some of the rules may well be committed to memory as containing points of the greatest value and importance to those engaged in nursing the sick. The book opens with some excellent remarks on a nurse's duties towards (1) herself, (2) her superiors, (3) her colleagues, (4) her patients. Following this a considerable space is devoted to the nursing of ordinary and extraordinary cases. Under the former heading such subjects as these are dealt with, viz., ventilation, bed-making, feeding and washing patients, the dressing of wounds, application of leeches, fomentations, &c. Some useful hints are given on the preparations necessary for post-mortem examination, which will be found of great service to the large number of nurses who are necessarily ignorant on the point. The treatment of bedsores is gone into at some length, though we confess to a wish that more prominence had been given to the necessity for thoroughly washing the back with a soapy lather night and morning as a protection against their formation. Spirits of wine are all very well as an addition when necessary, but the tendency is too often to use it as a substitute. Section 2, which deals with accidents and emergencies, contains much that will be found of the greatest practical value. Undressing the patient, for instance, requires much skill and forethought, so as to avoid the infliction of unnecessary pain. The remarks on hæmorrhage are clear, and admit of no misunderstanding. Operations also have a chapter to themselves, in which the chief points as regards the nursing are duly emphasised. Several excellent receipts are given under the heading of "Sick-room Cookery," which cannot fail to be of interest to the reader, variety being as great a difficulty as it is a necessity for the sick. The receipt for imperial drink should be known to every nurse as being the best way of preparing lemonade. The book concludes with a table of weights and measures and a glossary.

A SHORT MANUAL FOR MONTHLY NURSES. By CHARLES J. CULLINGWORTH, M.D., F.R.C.P. (Fourth Edition. London: J. and A. Churchill. 1896. Price 1s. 6d.)

This is a new edition of a well-known handbook. It has been revised, and in some parts much improved. Under the heading "Care of the Eyes," directions are given as to the mode of cleansing which is to be adopted if symptoms of purulent ophthalmia show themselves. The chapter on antiseptics appears to have been rewritten. It is to be noted that nurses are told that the antiseptic douche should be left to the discretion of the medical attendant, to be employed or not as he may direct. We find that sponges are referred to in various parts of the book as being used for purposes connected with the genitals. This we may suppose is a survival from old editions. At any rate, they are not mentioned in the list of articles needed. It would, however, have been better if the nurse had been definitely informed whether such things were to be used or not. The directions to the nurse as to what she should do in case the doctor does not arrive are, in one point, not altogether satisfactory. It does not seem to us a wise proceeding for the nurse (we are not here speaking of midwives, but of the sort of nurse for whom this manual is sufficient guide) to remove her hand from off the uterus in the third stage in order that she may fiddle about with the cord, and "hook down" the after-birth out of the vagina. Far better stick to the uterus and let the placenta alone. In general, however, the book is a reliable guide, and it is written throughout in a plain and simple style.

### Where to Go.

ST. CUTHBERT'S HALL, PHILBEACH GARDENS, S.W.—On Saturday, February 17th, under the patronage of the Duchess of Fife, a children's musical fairy play, called "Cinderella," will be given at this hall in aid of the Clarence Wing of St. Mary's Hospital, Paddington. The entertainment is under the direction of Miss Blanche Lawson, from whom tickets may be had at 224, Gloucester Terrace, Hyde Park.

CHILDREN'S HOME HOSPITAL, BARNET.—Two performances of "Sweet Lavender" will be given by well-known amateurs in aid of this useful little institution on Friday evening, February 19th, and Saturday afternoon, February 20th, at the Bijou Theatre, Westbourne Grove. Tickets, price 5s. and 2s. 6d., may be obtained from Mrs. Arthur Waugh, 11, Hillfield Road, West Hampstead.

THE DOWDESWELL GALLERIES, 160, NEW BOND STREET, W.—Messrs. Dowdeswell have a charming little exhibition on just now which well repays a visit, especially to those interested in landscape. Here Mr. J. Aumonier, R.I., has a series of fine pictures in oil, portraying some characteristic bits of scenery in Lincolnshire and Sussex, whilst in water colours we find a collection of drawings of the "Old Chain Pier, Brighton," painted in a sympathetic manner by another artist. These drawings portray the well-known scene in its every aspect, crowded and gay, deserted and sombre. They make an interesting collection, and show the Old Pier in a picturesque, romantic manner.

### For Reading to the Sick.

"GOD IS LOVE."

**Verses.**

ABOU BEN ADHEM (may his tribe increase !)
Awoke one night from a sweet dream of peace,
And saw, within the moonlight of his room,
Making it rich and like a lily in bloom,
An Angel, writing in a book of gold.
Exceeding peace had made Ben Adhem bold,
So to the Presence in the room he said :
"What writest thou?" The vision raised his head,
And, in a voice made of all sweet accord,
Answered : "The names of those who love the Lord !"
"And is mine one?" said Abou. "Nay, not so,"
Replied the Angel. Abou spake more low
And cheerly still, and said, "I pray thee, then,
Write me as one who loves his fellow-men."
The Angel wrote and vanished. The next night
He came again with a great wakening light,
And showed the souls whom love of God had blest,
And lo !—Ben Adhem's name led all the rest !

—*Leigh Hunt.*

Love is kind, and suffers long,
Love is meek, and thinks no wrong,
Love than death itself more strong ;
Therefore, give us love.

Prophecy will fade away,
Melting in the light of day ;
Love will ever with us stay ;
Therefore, give us love.

Faith will vanish into sight ;
Hope be emptied in delight ;
Love in Heaven will shine more bright ;
Therefore, give us love.

Faith and hope and love we see
Joining hand in hand agree ;
But the greatest of the three,
And the best is love.

—*Hymns Ancient and Modern.*

**Reading.**

"Beloved, let us love one and another : for love is of God ; and everyone that loveth is born of God, and knoweth God."

"He that loveth not, knoweth not God, for God is love."

And we have known and believed the love that God hath to us. God is love ; and he that dwelleth in love dwelleth in God, and God in him.

There is no fear in love ; but perfect love casteth out fear ; because fear hath torment. He that feareth is not made perfect in love.

We love Him, because He first loved us.

If a man say, I love God, and hateth his brother, he is a liar : for he that loveth not his brother whom he hath seen, how can he love God whom he hath not seen.—1 *St. John* vii. 20.

Who knows if love and its beatitude, clear manifestation as it is of the universal harmony of things, is not the best demonstration of a fatherly and understanding God, just as it is the shortest road by which to reach Him? Love is a faith, and one faith leads to another. And this faith is happiness, light, and force. Only by it does a man enter into the series of the living, the awakened, the happy, the redeemed—of those true men who know the value of existence, and who labour for the glory of God and of the truth. Till then we are but babblers and chatterers, spendthrifts of our time, our faculties, and our gifts, without aim, without real joy.

### Notes and Queries.

*Training.*

(135) Where could I obtain one year's general training before going in for midwifery training ?—*K. G.*

You can only get so short a period of training by paying for it. A good many hospitals take paying probationers either for periods of three months at a guinea a week, or for a year by paying a premium. For instance, at St. Mary's Hospital, Paddington, a year's training is given for a premium of £30. At the London Hospital and at Charing Cross, amongst others, the fee is thirteen guineas for three months. At the Soho Hospital for Women one year's training is given for a premium of forty guineas. For full details of the terms of training at London and provincial hospitals, see the lists in "How to Become a Nurse" (Scientific Press, 28 and 29, Southampton Street).

*A Book for Matrons.*

(136) Is there any book which gives hints on matrons' duties, amount per head for expenditure, and any other items of use to matrons ? If so, where can it be got, and what is the price ?—*Matron.*

You will find what you want in the series of articles on "Hospital Expenditure: The Commissariat," which began in THE HOSPITAL for October 5th, 1895, and is still continuing. In these all housekeeping questions, cost of provisions, &c., are dealt with fully, and we think would prove just what you want. You can obtain these numbers of THE HOSPITAL from the Manager, at the office. A practical way of getting hints on the duties of a matron when first taking up that important position is to consult other matrons, whose personal experience on many points will be of more value than any book.

*Nursing in India.*

(137) I am anxious to go to India to nurse. What steps can I take to ensure getting work there, and to whom could I apply for information ? —*Nurse Maude.*

Pray do not think of going out to India unless you have some sure and certain appointment awaiting you there. You could apply at the India Office for a form of application for the Indian Nursing Service, and see if your qualifications render you eligible, or you might apply to Lady Dufferin's Fund, 1, Victoria Street, E.C., by which nurses are sometimes sent out to India. There is Lady Roberts' Fund for Supplying Lady Nurses, but Lady Roberts selects her own nurses, and can only be approached by private introduction. You might write to Mrs. Nisbet, Madras General Hospital, and ask her advice as to private nursing prospects in India.

*Indiarubber Boots.*

(138) Referring to your recent article on "Cold Feet," will you kindly tell me where indiarubber boots as described can be obtained ?—*J. W.*

You can get snow boots of felt or indiarubber at any large boot shop.

*Training.*

(139) Will you tell me what qualifications are required of a probationer ? I am twenty-five years of age, and have made many applications, and, after filling up the forms, been refused without any reason given.—*E. Sheffield.*

Many would-be candidates fill up the forms of application sent to them by the matrons to whom they apply without carefully reading the printed regulations and noting if they are really eligible, and so cause disappointment to themselves and give needless trouble. You may have many disqualifications which you do not mention in your letter which may account for your non-success. Very good health is required of probationers, and the general standard of education is far higher now than it used to be. Also, so many women wish to train as nurses nowadays that matrons can afford in many instances to take their choice of the candidates who apply for vacancies. If you are really well suited for nursing and persevere in your efforts to obtain training you are certain to succeed. You should apply at some of the smaller provincial hospitals where the vacancies are not in so much demand, or at the infirmaries.

*Middlesex Hospital Journal.*

(140) Where can I procure a copy of this publication, which is mentioned in this week's HOSPITAL ?

The "Middlesex Hospital Journal" is published by Mitchell and Hughes, 140, Wardour Street, W., price 1s.

*Continental Tours.*

(141) Can you give me any information about cheap Continental tours which a nurse could join ? I hear the London Polytechnic arranges tours. Would it be possible to avail oneself of these, and how could I get to know ?—*Ingaborg.*

Write to the London Polytechnic, Langham Place, W., asking for particulars of their tours. They are, we believe, very well managed, and you would almost certainly find some pleasant travelling companions amongst those going. You should try to find some friend or acquaintance to go at the same time, which would add very greatly to the pleasure of the trip. You will find useful information on matters of travel in the columns of the *Queen* devoted to that special subject each week.

*A Cookery Book.*

(142) Can you recommend me a good cookery book in which I may find some easy tasty dishes for a great invalid ?—*Nurse E. B.*

Get "The Art of Cooking for Invalids," by Florence B. Jack, price 2s. (Whittaker and Co., Paternoster Row.)

*Voluntary and State Institutions.*

(143) Are general hospitals under the Local Government Board, or only the workhouses ?—*E. H.*

Of course the general hospitals which are supported by voluntary contributions are not under the L.G.B. The large separate infirmaries attached to the various unions are rate-supported and so under the control of the L.G.B. in the same way that the workhouses and the sick wards in the smaller workhouses are.

